# Heritability of Body Mass Index Among Familial Generations

**DOI:** 10.1001/jamanetworkopen.2024.19029

**Published:** 2024-06-28

**Authors:** Gabriel Chodick, Maya Simchoni, Britt Wang Jensen, Estela Derazne, Orit Pinhas-Hamiel, Regev Landau, Alon Abramovich, Arnon Afek, Jennifer Lyn Baker, Gilad Twig

**Affiliations:** 1 Department of Epidemiology and Preventive Medicine, Faculty of Medicine, Tel Aviv University, Tel Aviv, Israel; 2 Israel Defense Forces Medical Corps, Ramat Gan, Israel; 3 Department of Military Medicine, Hebrew University, Jerusalem; 4 Center for Clinical Research and Prevention, Copenhagen University Hospital–Bispebjerg and Frederiksberg, Copenhagen, Denmark; 5 Faculty of Medicine, Tel Aviv University, Tel Aviv, Israel; 6 Pediatric Endocrine and Diabetes Unit, Edmond and Lily Safra Children’s Hospital, Sheba Medical Center at Tel Hashomer, Ramat Gan, Israel; 7 Central Management, Sheba Medical Center at Tel Hashomer, Ramat Gan, Israel; 8 The Institute of Endocrinology, Diabetes and Metabolism, Sheba Medical Center at Tel Hashomer, Ramat Gan, Israel; 9 The Gertner Institute for Epidemiology & Health Policy Research, Sheba Medical Center at Tel Hashomer, Ramat Gan, Israel

## Abstract

**Importance:**

Studies on the familial effects of body mass index (BMI) status have yielded a wide range of data on its heritability.

**Objective:**

To assess the heritability of obesity by measuring the association between the BMIs of fathers, mothers, and their offspring at the same age.

**Design, Setting, and Participants:**

This cohort study used data from population-wide mandatory medical screening before compulsory military service in Israel. The study included participants examined between January 1, 1986, and December 31, 2018, whose both parents had their BMI measurement taken at their own prerecruitment evaluation in the past. Data analysis was performed from May to December 2023.

**Main Outcomes and Measures:**

Spearman correlation coefficients were calculated for offsprings’ BMI and their mothers’, fathers’, and midparental BMI percentile (the mean of the mothers’ and fathers’ BMI cohort- and sex-specific BMI percentile) to estimate heritability. Logistic regression models were applied to estimate the odds ratios (ORs) and 95% CIs of obesity compared with healthy BMI, according to parental BMI status.

**Results:**

A total of 447 883 offspring (235 105 male [52.5%]; mean [SD] age, 17.09 [0.34] years) with both parents enrolled and measured for BMI at 17 years of age were enrolled in the study, yielding a total study population of 1 343 649 individuals. Overall, the correlation between midparental BMI percentile at 17 years of age and the offspring’s BMI at 17 years of age was moderate (ρ = 0.386). Among female offspring, maternal-offspring BMI correlation (ρ = 0.329) was somewhat higher than the paternal-offspring BMI correlation (ρ = 0.266). Among trios in which both parents had a healthy BMI, the prevalence of overweight or obesity in offspring was 15.4%; this proportion increased to 76.6% when both parents had obesity and decreased to 3.3% when both parents had severe underweight. Compared with healthy weight, maternal (OR, 4.96; 95% CI, 4.63-5.32), paternal (OR, 4.48; 95% CI, 4.26-4.72), and parental (OR, 6.44; 95% CI, 6.22-6.67) obesity (midparent BMI in the ≥95th percentile) at 17 years of age were associated with increased odds of obesity among offspring.

**Conclusions and Relevance:**

In this cohort study of military enrollees whose parents also underwent prerecruitment evaluations, the observed correlation between midparental and offspring BMI, coupled with a calculated narrow-sense heritability of 39%, suggested a substantive contribution of genetic factors to BMI variation at 17 years of age.

## Key Points

**Question:**

What is the association between parents’ body weight at 17 years of age and the likelihood of offspring obesity, accounting for the influence of shared environmental and lifestyle factors?

**Findings:**

In this cohort study of more than 1.3 million individuals, offspring born to parents with obesity at 17 years of age exhibited a substantial 77% probability of having obesity at the same age compared with 15% when both parents were in the healthy weight range.

**Meaning:**

In this study, the weight status of parents at 17 years of age was associated with obesity risk for both female and male offspring, emphasizing that parental factors may influence the next generation’s health outcomes.

## Teaser

This cohort study examines the association of offsprings’ body mass index at age 17 years with the body mass index of their parents at the same age.

## Introduction

Childhood obesity has emerged as a pressing global health concern, with its prevalence steadily increasing during the past several decades.^1^ This alarming trend has led researchers to delve deeper into the multifaceted factors contributing to this epidemic, including the potential association between parental body mass index (BMI) and the development of obesity in their offspring. Genetic factors play a pivotal role in determining an individual’s growth and height potential. When examining nuclear families, the accuracy of estimating final height potential exceeds 95% when using midparental height as a key metric.^2^ In contrast, much lower heritability levels are measured for body weight.^3,4,5^

One explanation for the discrepancy in heritability between height and body weight may be attributed to the dynamic nature of weight changes throughout adulthood, suggesting the progressively increasing importance of nongenetic factors.^6^ Consequently, cross-sectional family studies, particularly those involving parents spanning a wide range of middle to late adulthood ages, might underestimate the complete scope of body weight heritability.

This cross-generational cohort study aims to examine the association between parental BMI and offspring obesity while minimizing the effect of shared environmental and lifestyle choices within the family unit. We used the national database of Israeli army recruits to examine the association between parental and offspring BMI, all measured at 17 years of age, before the parents could have a shared family household. Our hypothesis is that assessing parental and offspring’s BMI at a similar age, rather than at a similar time, would show higher heritability.

## Methods

The Israel Defense Forces (IDF) Medical Corps Institutional Review Board approved this study and waived the requirement for informed consent because data confidentiality was highly protected in this large database analysis. This study followed the Strengthening the Reporting of Observational Studies in Epidemiology (STROBE) reporting guideline.

### Study Population

The Security Service Act mandates that all Jewish citizens of Israel serve in the IDF at 18 years of age (except those who qualify for exemptions from service, such as ultraorthodox communities). The law authorizes the secretary of defense to summon citizens for mandatory medical screenings 1 year before their service to determine conscription eligibility and assess their medical fitness for different military roles. Included in this analysis were all military enrollees whose fathers and mothers also underwent prerecruitment evaluations, including review of medical history, health examinations, and weight and height measurements (complete trios) between January 1, 1986, and December 31, 2018. Data on height or weight measurements were missing in 3% of the 1.9 million individuals who underwent prerecruitment evaluation during the study period. More than 50% of the parents included in the study are Jewish immigrants from various geographic areas, providing a broad and diverse representation of the general population.

### BMI Measurements and Other Study Variables

We calculated the BMI as weight in kilograms divided by height in meters squared using weight and height measured by means of a beam balance and stadiometer to the nearest 0.1 kg and 1.0 cm, respectively, with each participant barefoot and wearing underwear. Sociodemographic variables, including residential sociodemographic status (SES) and years of education, were reported to military authorities by governmental agencies as part of prerecruitment evaluation.^7^ Cognitive performance was also routinely assessed by a cognitive test that included 4 subsets (a modified Otis-type verbal intelligence test, which measures ability to understand and perform verbal instructions; a modified version of the similarities subtest of the Wechsler intelligence scales, which assesses verbal abstraction and categorization; mathematical reasoning, concentration, and concept manipulation; and a modified version of Raven progressive matrices, which measures nonverbal abstract reasoning and visual-spatial problem-solving abilities). The summed score of all tests together, ranging from 10 to 90 points (with 10 indicating the lowest cognitive performance and 90 indicating the highest, forms a validated measure of general intelligence^8^ and is strongly negatively associated with metabolic outcomes.^9,10^

### Statistical Analysis

The age at the date of examination, year of birth, and height were treated as continuous variables and expressed as mean (SD). Educational level was categorized based on whether the participant attended formal schooling for less than 11 years or 11 to 12 years (the maximum possible at point of assessment). Socioeconomic status was estimated according to residential locality,^11^ with participants grouped into low, medium, and high categories. To adjust for the temporal increase in BMI by calendar years, we transformed BMI into sex- and year of assessment–specific percentiles based on the data included in this study. The BMI percentiles were classified as severely underweight (<3rd percentile), healthy weight (3rd to <85th percentile), overweight (85th to <95th percentile), and obesity (≥95th percentile). We calculated Spearman correlation coefficients between the offsprings’ BMI and that of their mothers and fathers. We also calculated coefficients for offspring BMI regressed on midparental BMI percentile (the mean of the mother’s and father’s BMI) to estimate heritability.^12^ Analyses were stratified for male and female participants separately. We focused on Spearman correlation coefficients because they do not assume normality and are conservative when linearity assumptions hold. Analyses were further stratified by year of BMI assessment, SES quintile, and number of siblings who had a prerecruitment evaluation in the past. To assess potential confounding by coexisting illness, we also restricted the subanalyses to participants with unimpaired health by excluding those with a history of morbidity that requires long-term medical treatment or follow-up, including any major surgery or cancer. One potential path that could contribute to the heritability of obesity is assortative mating, when mate selection is influenced by body size.^3^ To assess the potential influence of body size, the correlation between offspring’s maternal and paternal BMI was also calculated.

Prevalence and Fisher exact test 95% CI estimates were determined for severely underweight, healthy weight, overweight, and obesity at the age of approximately 17 years. Bivariate and multivariable logistic regression models were applied to estimate the odds ratios (ORs) and 95% CIs of overweight or obesity compared with healthy weight according to parental BMI status. Two adjustment levels were applied. Model 1 adjusted for offspring’s age at BMI measurement and sex. Model 2 additionally adjusted for calendar year of BMI measurement (as a continuous variable), socioeconomic level (low, medium, or high), educational level (<11 or 11-12 formal schooling years), cognitive performance (low, medium, or high tertile), and father’s and mother’s BMI category (eTable 2 in Supplement 1). To examine whether offspring’s obesity varied independently across paternal and maternal BMI levels, we tested their interaction product terms in the regression model. All tests were 2-sided, and the significance level was set as *P* < .05. The main analyses were performed with the use of SPSS software, version 25.0 (IBM Inc) and R studio, version 2024.04 (R Project for Statistical Computing). Data analysis was performed from May to December 2023.

## Results

We identified a total of 447 883 offspring (235 105 male [52.5%] and 212 778 female [47.5%]) with both parents enrolled, yielding a total study population of 1 343 649 individuals. Overall, the total number of eligible examinees accounts for 23.8% of all individuals who underwent prerecruitment assessment during the study period (eFigure 1 in Supplement 1). A total of 408 262 participants (47.5%) were born in Israel, 17 336 (2.0%) were born in the former Soviet Union, 136 920 (15.9%) were born in Asia, 111 938 (13.0%) were born in Africa, and 184 363 (21.5%) were born in Europe or the US. Although generally similar characteristics were observed between the included and excluded individuals across sex and examination periods, some difference (standardized mean difference, ≤0.58) in SES and cognitive performance were observed (eTable 1 in Supplement 1). The mean (SD) age at BMI measurement among offspring and their parents was 17.09 (0.34) and 17.43 (0.31) years, respectively (Table 1). Height was comparable between female offspring (162.20 [6.15] cm) and their mothers (162.08 [5.86] cm) as well as among male offspring (174.62 [6.72] cm) and their fathers (174.01 [6.45] cm). In contrast, body weight was substantially higher among offspring compared with their parents, both among female offspring and their mothers (58.18 [11.04] vs 56.10 [8.32] kg; standardized mean difference, 0.21) and in male offspring and their fathers (68.25 [13.12] vs 64.88 [9.85] kg; standardized mean difference, 0.29).

**Table 1.** Demographic Characteristics of the Participants and Both Parents, All Measured at 17 Years of Age^a^

Characteristic	Male	Female	Total
**Offspring**			
No.	235 105	212 778	447 883
Age, mean (SD), y	17.13 (0.35)	17.05 (0.32)	17.09 (0.34)
Year of birth, median (range)	1993 (1973-2002)	1993 (1973-2002)	1993 (1973-2002)
BMI, mean (SD)	22.34 (3.91)	22.09 (3.85)	22.22 (3.88)
Height, mean (SD), cm	174.62 (6.72)	162.20 (6.15)	168.72 (8.96)
Weight, mean (SD), kg	68.25 (13.12)	58.18 (11.04)	63.46 (13.17)
Unimpaired health	161 216 (68.6)	144 720 (68.0)	305 936 (68.3)
Education, y			
<11	6375 (2.7)	1727 (0.8)	8102 (1.8)
≥11	228 420 (97.3)	210 801 (99.2)	439 221 (98.2)
SES level			
1-4 (lowest)	34 297 (14.7)	27 634 (13.0)	61 931 (13.9)
5-7	128 940 (55.1)	117 299 (55.4)	246 239 (55.2)
8-10	70 820 (30.3)	66 950 (31.6)	137 770 (30.9)
Cognitive performance, points			
10-30	28 239 (12.0)	21 062 (9.9)	49 301 (11.0)
40-70	159 360 (67.8)	163 969 (77.1)	323 329 (72.2)
80-90	47 506 (20.2)	27 747 (13.0)	75 253 (16.8)
**Parents**			
No.	447 883	447 883	895 766
Age, mean (SD), y	17.39 (0.28)	17.47 (0.34)	17.43 (0.31)
Year of birth, median (range)	1960 (1947-1982)	1964 (1948-1984)	1962 (1947-1984)
BMI, mean (SD)	21.39 (2.77)	21.34 (2.82)	21.36 (2.8)
Height, mean (SD), cm	174.01 (6.45)	162.08 (5.86)	168.04 (6.15)
Weight, mean (SD), kg	64.88 (9.85)	56.10 (8.32)	60.49 (9.08)
Unimpaired health	366 776 (81.9)	367 305 (82.0)	734 081 (82.0)
Education, y			
<11	109 999 (24.56)	45 557 (10.17)	155 556 (17.37)
≥11	337 845 (75.43)	402 269 (89.82)	740 114 (82.62)
SES level			
1-4 (Lowest)	92 083 (20.56)	87 849 (19.61)	179 932 (20.09)
5-7	237 744 (53.08)	231 108 (51.6)	468 852 (52.34)
8-10	113 567 (25.36)	113 943 (25.44)	227 510 (25.4)
Cognitive performance, points			
10-30	63 709 (14.22)	88 270 (19.71)	151 979 (16.97)
40-70	281 934 (62.95)	287 916 (64.28)	569 850 (63.62)
80-90	102 240 (22.83)	71 697 (16.01)	173 937 (19.42)
Geographic regions			
Israel	199 353 (46.5)	208 909 (48.6)	408 262 (47.5)
Former Soviet Union	9322 (2.2)	8014 (1.9)	17 336 (2.0)
Asia	68 521 (16.0)	68 399 (15.9)	136 920 (15.9)
Africa	55 707 (13.0)	56 231 (13.1)	111 938 (13.0)
Europe or US	96 003 (22.4)	88 360 (20.6)	184 363 (21.5)

Abbreviations: BMI, body mass index (calculated as weight in kilograms divided by height in meters squared); SES, socioeconomic status.

^a^ Data are presented as number (percentage) unless otherwise indicated.

**Figure 1.**
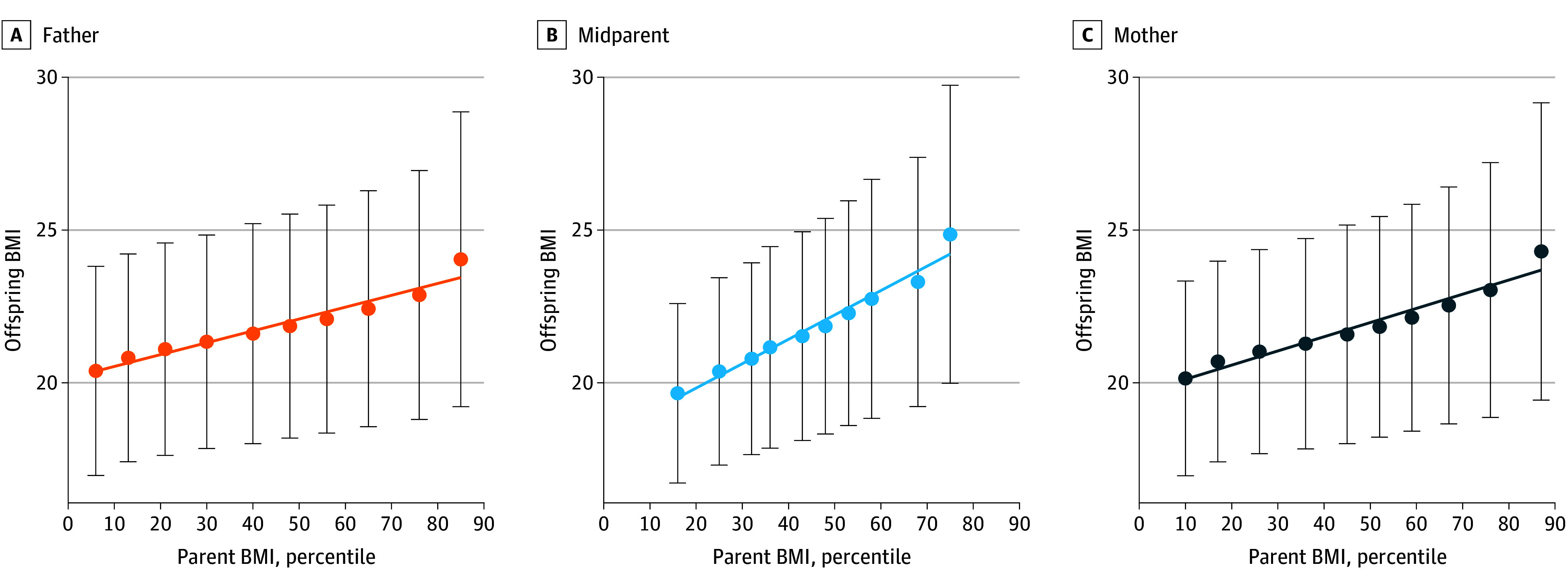
Mean Offspring Body Mass Indexes (BMI) According to Age- and Cohort-Adjusted Deciles of Father’s, Mother’s, and Midparental BMI Percentile Analyses are based on 447 883 complete parent-offspring trios taken at military prerecruitment examinations (at approximately 17 years of age). The BMIs were calculated as weight in kilograms divided by height in meters squared. Error bars indicate SDs.

Figure 1 depicts offspring BMI according to parental and midparental BMI decile. The correlations as well as parental pair associations are presented in Table 2. Overall, the correlation between midparental BMI percentile at 17 years of age and offsprings’ BMI at 17 years of age was moderate (ρ = 0.386), suggesting a narrow-sense heritability for the additive genetic variance in BMI of 39%. Among female participants, the maternal-offspring BMI correlation (ρ = 0.329) was somewhat higher than the paternal-offspring BMI correlation (ρ = 0.266). In male participants, the paternal-offspring BMI correlation (ρ = 0.273) and maternal-offspring BMI correlation (ρ = 0.278) were comparable (Table 2). Overall, midparental BMI showed a greater correlation with offspring BMI compared with maternal or paternal BMI. Results remained robust across offspring year of BMI measurement, offspring health status, and number of siblings. The correlation between maternal and paternal BMI was weak (ρ = 0.069).

**Table 2.** Spearman Correlation Coefficients Between Parental and Offspring BMI Percentiles at 17 Years of Age^a^

Offspring	Spearman ρ coefficient		
	Father	Mother	Midparent
Total (N = 447 883)	0.269	0.301	0.386
Sex			
Male (n = 235 105)	0.273	0.278	0.372
Female (n = 212 778)	0.266	0.329	0.402
Year of BMI measurement			
1980-1989 (n = 298)	0.278	0.221	0.320
1990-1999 (n = 53 219)	0.268	0.300	0.380
2000-2009 (n = 160 439)	0.276	0.305	0.389
2010-2018 (n = 233 927)	0.283	0.303	0.385
Health status at age 17 y			
Unimpaired (n = 305 936)	0.297	0.324	0.416
Impaired (n = 141 947)	0.254	0.288	0.368
No. of siblings			
0 (n = 368 381)	0.269	0.300	0.385
≥1 (n = 61 502)	0.275	0.305	0.391
SES			
0 (Lowest, n = 21 442)	0.284	0.298	0.392
1-2 (n = 27 520)	0.279	0.309	0.397
3-4 (n = 54 823)	0.285	0.316	0.403
5-6 (n = 76 470)	0.277	0.303	0.392
7-8 (n = 103 564)	0.269	0.298	0.384
9-10 (Highest, n = 128 221)	0.257	0.290	0.370

Abbreviations: BMI, body mass index (calculated as weight in kilograms divided by height in meters squared); SES, socioeconomic status.

^a^ All Spearman correlation coefficients are statistically significant (*P* < .001).

**Figure 2.**
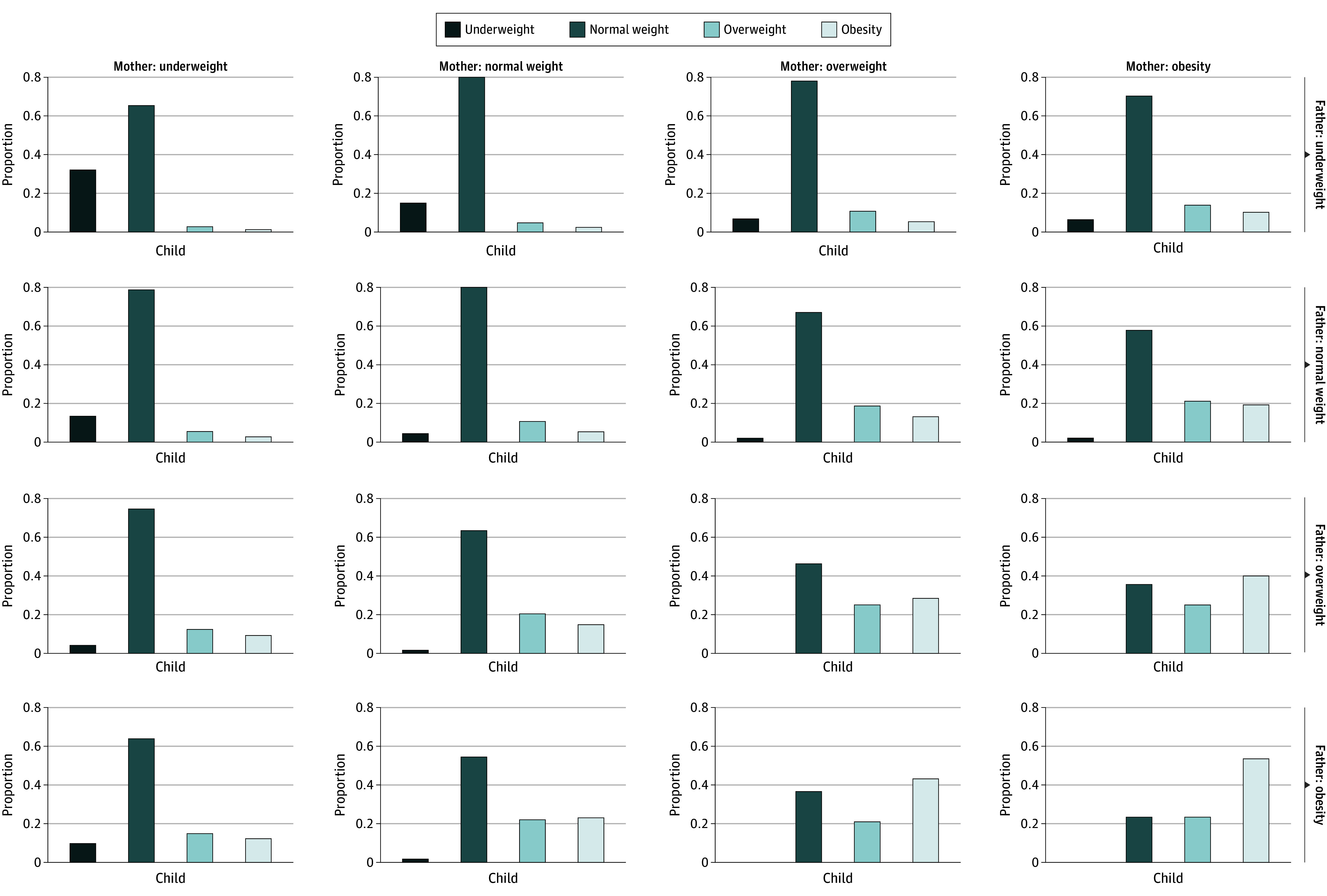
Mean Body Mass Index (BMI) Distribution of Offspring According to Maternal and Paternal BMI Status Analyses are based on 447 883 complete parent-offspring (of both sexes) trios taken at military prerecruitment examinations (at approximately 17 years of age). The BMIs were calculated as weight in kilograms divided by height in meters squared.

The distribution of offspring BMI status according to maternal and paternal BMI percentile is depicted in Figure 2. Among trios in which both parents were classified as having a healthy BMI at 17 years of age (constituting 60% of the study trios), the prevalence of overweight or obesity (BMI ≥85th percentile) in offspring was 15.4% (95% CI,15.2%-15.5%). This prevalence increased to 76.6% (95% CI, 71.8%-80.8%) when both parents had obesity and decreased to 3.3% (95% CI, 1.8%-3.6%) when both parents had severe underweight.

**Figure 3.**
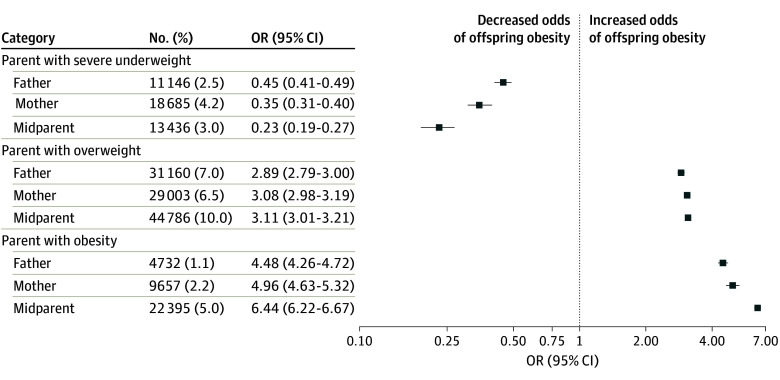
Adjusted Odds Ratios (ORs) for Obesity in Offspring by Parental Body Mass Index (BMI) Status Compared With Parents With Healthy BMI Range In all trios, the BMI was measured at 17 years of age. The ORs for fathers’ and mothers’ BMI status were adjusted for the other parent BMI status. The ORs are adjusted for offspring’s age, sex, year of BMI measurement, socioeconomic level, educational level, cognitive performance, and father’s and mother’s BMI category. The BMIs were calculated as weight in kilograms divided by height in meters squared. Error bars indicate 95% CIs. See eTable 6 in Supplement 1 for details.

Figure 3 and eTable 2 in Supplement 1 show the univariate and multivariable logistic regression models of the odds of obesity in children and adolescents by parental BMI status at 17 years of age. Compared with healthy weight, paternal (OR, 4.48; 95% CI, 4.26-4.72), maternal (OR, 4.96; 95% CI, 4.63-5.32), and parental (OR, 6.44; 95% CI, 6.22-6.67) obesity (midparent BMI ≥95th percentile) at 17 years of age were associated with increased odds of obesity among offspring. Somewhat stronger associations were found between parental obesity and obesity in female offspring (eTable 3 and eFigure 2 in Supplement 1) compared with male offspring (eTable 4 and eFigure 3 in Supplement 1). No significant interactions were identified between specific parental BMI levels, with the exception of mothers with overweight, with a positive interaction when fathers had severe underweight and a negative interaction when fathers had overweight or obesity (eTable 5 in Supplement 1). Having a parent with severe underweight was negatively associated with the likelihood of having obesity compared with parents with healthy BMI, accounting for the other parent’s BMI (OR, 0.45; 95% CI, 0.41-0.49 and OR, 0.35; 95% CI, 0.31-0.40 for father’s and mother’s underweight, respectively).

## Discussion

In this large, population-based cohort, we studied 447 883 mother-father-offspring trios, each systematically assessed for BMI during their military recruitment around the age of 17 years. Our investigation revealed a noteworthy correlation of 0.386 between midparental and offspring BMI, indicating a moderate association. The estimation of narrow-sense heritability for the additive genetic variance in BMI stood at 39%.

Comparative analyses with other studies underscored the robustness of our findings. Our estimated narrow-sense heritability is similar to a pooled estimate of 0.42 in a meta-analysis^13^ of 25 family studies comprising 42 968 individuals from diverse populations. Notably, our observed correlation surpassed those documented in the Québec Family Study (ρ = 0.23)^14^ and the National Health and Nutrition Examination Survey III (ρ = 0.27)^15^ for parental and adolescent or young adulthood offspring BMI centiles. Aligning with our results, a longitudinal examination spanning 2 decades within the Framingham Heart Study, limited to individuals aged 30 to 40 years, exhibited heritability estimates of 37%.^16^ No significant additive genetic contribution was found in the correlations between parental and offspring BMI in our study across calendar year, the number of siblings, and socioeconomic status.

The correlation in father-son BMI centile in our study (ρ = 0.273) closely aligns with that found among fathers and sons in Sweden’s Military Service Conscription Register (ρ = 0.28),^17^ emphasizing the consistency of our results with international datasets. Much higher heritability estimates for BMI were found in a study comparing pairs of monozygotic and dizygotic twins (ρ = 0.5-0.9),^3^ suggesting a substantial influence of genetic factors on BMI variation. However, in addition to the level of genetic differences, pairs of monozygotic twins also have a greater resemblance in prenatal and postnatal environmental conditions compared with dizygotic twins, which may have inflated the true heritability of BMI.^3^

Interestingly, we found a stronger correlation in BMI percentile between mothers and daughters compared with mothers and sons. Similarly, maternal obesity was associated with higher odds of obesity in female offspring compared with paternal obesity. This finding not only underscores the complexity of genetic and environmental interactions but also may suggest potential sex-specific influences on the heritability of BMI.^18^

Our prospective data from a population-based cohort revealed that maternal obesity and paternal obesity were profound risk factors for offspring obesity at 17 years of age, even after adjustment for potential confounding factors. Similar associations were reported in the Western Australian Pregnancy Cohort (Raine) Study in which maternal and paternal obesity at late adolescence were associated with ORs of 6.96 and 3.75, respectively, for offspring obesity at 22 years of age.^19^ This parallel finding across distinct cohorts reinforces the consistency and generalizability of the observed intergenerational association of parental obesity with offspring weight status. Our findings of BMI of offspring being higher than that of their parents at 17 years of age are consistent with previous studies.^18,20^

Notably, our investigation aimed to explore the combined influence of both parents using midparental BMI percentile. Parental obesity did not exhibit a notable multiplicative or additive synergy concerning the likelihood of offspring obesity. These nuanced insights contribute to a more comprehensive understanding of the intricate interplay between parental obesity and its association with the weight status of the next generation.

### Strengths and Limitations

Our study has several important strengths. The use of a population-based cohort comprising 447 883 mother-father-offspring trios and the constancy of the estimated correlations across different SES levels, time periods, and baseline medical status enhances the generalizability of our findings. More than 50% of the parents included in the study are Jewish immigrants from various geographic areas and are considered to possess highly diverse genetics, providing a broad and diverse representation of the general population. The large sample size provides high statistical power because the sampling variance of the estimate of heritability is inversely proportional to the association of number of individuals squared. In previous cross-sectional studies, confounding might lead to severe bias in the estimate of heritability. For example, if the resemblance of parents and offspring is partly due to common environmental effects, then an estimate of heritability that is based on their resemblance will be biased upward. In our case, the nuclear family had not been established when parents were measured, reducing the level of bias. The denominator of heritability is the total phenotypic variance, which has been assessed after full correction for known fixed effects by using sex-, age-, and cohort-specific BMI percentile. Other study strengths include a long period of observation spanning more than 3 decades of military recruits, systematic data collection including weight and height measurements in unselected population, and careful assessment of health status at adolescence.

Dissecting the factors that affect familial resemblance in BMI can be challenging, given the potential confounding effects of cultural transmission and shared environmental factors. To mitigate these complexities, a strategic approach, akin to the one used in the Framingham study^16^ and replicated in our analysis, involved imposing a restricted age range for BMI measurements to limit the influence of the familial environment and the influence of factors that may be age dependent.

These factors were further restricted by using BMI at 17 years of age, before establishing the shared household. This deliberate choice helped isolate and elucidate the genetic and environmental influences on BMI, unrestricted by the immediate familial environment. By anchoring our analysis in this critical timeframe, we sought to disentangle the intricate interplay of genetic and environmental factors, contributing to a more nuanced understanding of the dynamics shaping familial resemblance in BMI.

Our study also has limitations. Notably, potential biases and confounding factors need consideration. Specifically, parental educational level was in many studies,^21^ but not in all,^22^ inversely correlated with offspring obesity. Because our analysis was confined to late adolescence, it inherently lacked the ability to capture high levels of education. Although this limitation could introduce bias, it is noteworthy that our data exhibited only marginal differences in correlation coefficients between parental and offspring BMI percentiles across SES categories, which are often closely associated with educational levels. This finding suggests that the residual confounding effect stemming from higher education is likely minimal.

Assortative mating and selection are well-recognized sources of bias and misclassification in heritability studies.^23^ However, we found little evidence of assortative mating, with only a weak correlation between fathers’ and mothers’ BMI percentiles. Nonpaternity events, a pervasive concern in such studies, were addressed through an examination of the strength of paternal-offspring associations. According to a previous study, the prevalence of nonpaternity event ranges from less than 1% to more than 10%.^24^ Our findings, which did not exhibit a notable divergence between father-son and mother-son associations, suggest that nonpaternity is unlikely to introduce significant bias into our results. In addition, we lacked information on physical activity and diet. The role of these common environmental factors on BMI is substantial in young children but weakens or disappears at adolescence.^6,18,19^ Our data on number of siblings were limited to elder brothers and sisters who previously underwent prerecruitment. These missing data items, however, are not expected to arise from differential bias regarding the analysis presented. Also, all body weight measurements in this study were taken once at late adolescence, preventing the ability to assess heritability over time or to fully address the influence of era. Specifically, it was suggested that the increasingly obesogenic environment during recent decades may amplify genetic risk of obesity.^25^ Nonetheless, our correlation estimates for parental-offspring BMI showed only little variation during the 4-decade study period.

We had trio data available for only 24% of the entire population, primarily due to the continuous immigration to Israel during the study period. According to the Israeli Census for 2002^26^ (midpoint for year of offspring measurement), 37% of Jewish adults at the relevant parental age (20-55 years) were born outside Israel. In addition, changes in the mandatory recruitment policy for ultraorthodox men and women may have contributed to trio data completeness. Excluded individuals were somewhat more likely to belong to lower SES level and have lower cognitive scores. Nonetheless, the intergenerational correlation of BMI showed only small variation across levels of SES; thus, the exclusion of these individuals was unlikely to influence the validity of study results. Finally, the reliance on BMI as a surrogate measure for adiposity is acknowledged as a limitation. Although widely used, BMI has well-documented imperfections, especially in assessing abdominal obesity,^27^ a potent variable in the development of future noncommunicable chronic diseases.^28^ Waist circumference is not measured among recruits to the IDF and thus unavailable for our analysis, but it was previously shown that both maternal and paternal waist circumference is associated with a higher waist circumference of their young adult offspring, with a substantially greater regression coefficient compared with the correlation in BMI.^29^

## Conclusions

Our examination of familial resemblance in BMI in late adolescence within a large population-based cohort attempted to quantify the genetic component influencing body weight at 17 years of age. The observed moderate correlation between midparental and offspring BMI, coupled with a calculated narrow-sense heritability of 39%, suggests a substantive contribution of genetic factors to BMI variation. The links established between parental and offspring obesity, particularly during late adolescence, provide insights for understanding the early origins of obesity. The lack of significant additive or multiplicative synergy between parental obesity further refines our understanding of the dynamics shaping obesity risk.

## Notes

Supplement 1. **eFigure 1.** Attrition Chart of Study Population **eFigure 2.** Odds Ratio for Female Offspring Obesity at Age 17, According to Father's, Mother's, and Mid-Parental BMI Status, Compared to Parent in Normal BMI Range **eFigure 3.** Odds Ratio for Male Offspring Obesity at Age 17, According to Father's, Mother's, and Mid-Parental BMI Status, Compared to Parent in Normal BMI Range **eTable 1.** Characteristics of Those Included and Excluded From the Analysis, by Sex and Period **eTable 2.** Multivariable Logistic Regression for Obese Offspring According to Parents' BMI **eTable 3.** Multivariable Logistic Regression for Offspring With Obesity According to Parents’ BMI, Males **eTable 4.** Multivariable Logistic Regression for Offspring With Obesity According to Parents' BMI, Females **eTable 5.** Logistic Regression Model: Interaction Terms of Parental BMI Levels **eTable 6.** List of Variables in the Multivariable Logistic Regression Shown in eTable 1

Supplement 2.
Data Sharing Statement
