# Supplementary material for: Heritability of Body Mass Index Among Familial Generations
Source: JAMA Netw Open. 2024 Jun 28;7(6):e2419029. doi: 10.1001/jamanetworkopen.2024.19029 (PMC11214117; doi:10.1001/jamanetworkopen.2024.19029)
Supplement: Supplement 1. — eFigure 1. Attrition Chart of Study Population eFigure 2. Odds Ratio for Female Offspring Obesity at Age 17, According to Father's, Mother's, and Mid-Parental BMI Status, Compared to Parent in Normal BMI Range eFigure 3. Odds Ratio for Male Offspring Obesity at Age 17, According to Father's, Mother's, and Mid-Parental BMI Status, Compared to Parent in Normal BMI Range eTable 1. Characteristics of Those Included and Excluded From the Analysis, by Sex and Period eTable 2. Multivariable Logistic Regression for Obese Offspring According to Parents' BMI eTable 3. Multivariable Logistic Regression for Offspring With Obesity According to Parents’ BMI, Males eTable 4. Multivariable Logistic Regression for Offspring With Obesity According to Parents' BMI, Females eTable 5. Logistic Regression Model: Interaction Terms of Parental BMI Levels eTable 6. List of Variables in the Multivariable Logistic Regression Shown in eTable 1 [file jamanetwopen-e2419029-s001.pdf]

Supplementary Online Content

Chodick G, Simchoni M, Jensen BW, et al. Heritability of body mass index among multiple familial generations. *JAMA Netw Open*. 2024;7(6):e2419029. doi:10.1001/jamanetworkopen.2024.19029

eFigure 1. Attrition Chart of Study Population

eFigure 2. Odds Ratio for Female Offspring Obesity at Age 17, According to Father's, Mother's, and Mid-Parental BMI Status, Compared to Parent in Normal BMI Range

eFigure 3. Odds Ratio for Male Offspring Obesity at Age 17, According to Father's, Mother's, and Mid-Parental BMI Status, Compared to Parent in Normal BMI Range

eTable 1. Characteristics of Those Included and Excluded From the Analysis, by Sex and Period

eTable 2. Multivariable Logistic Regression for Obese Offspring According to Parents' BMI

eTable 3. Multivariable Logistic Regression for Offspring With Obesity According to Parents' BMI, Males

eTable 4. Multivariable Logistic Regression for Offspring With Obesity According to Parents' BMI, Females

eTable 5. Logistic Regression Model: Interaction Terms of Parental BMI Levels

eTable 6. List of Variables in the Multivariable Logistic Regression Shown in eTable 1

This supplementary material has been provided by the authors to give readers additional information about their work.

© 2024 Chodick et al. *JAMA Network Open.*

eFigure 1. Attrition Chart of Study Population

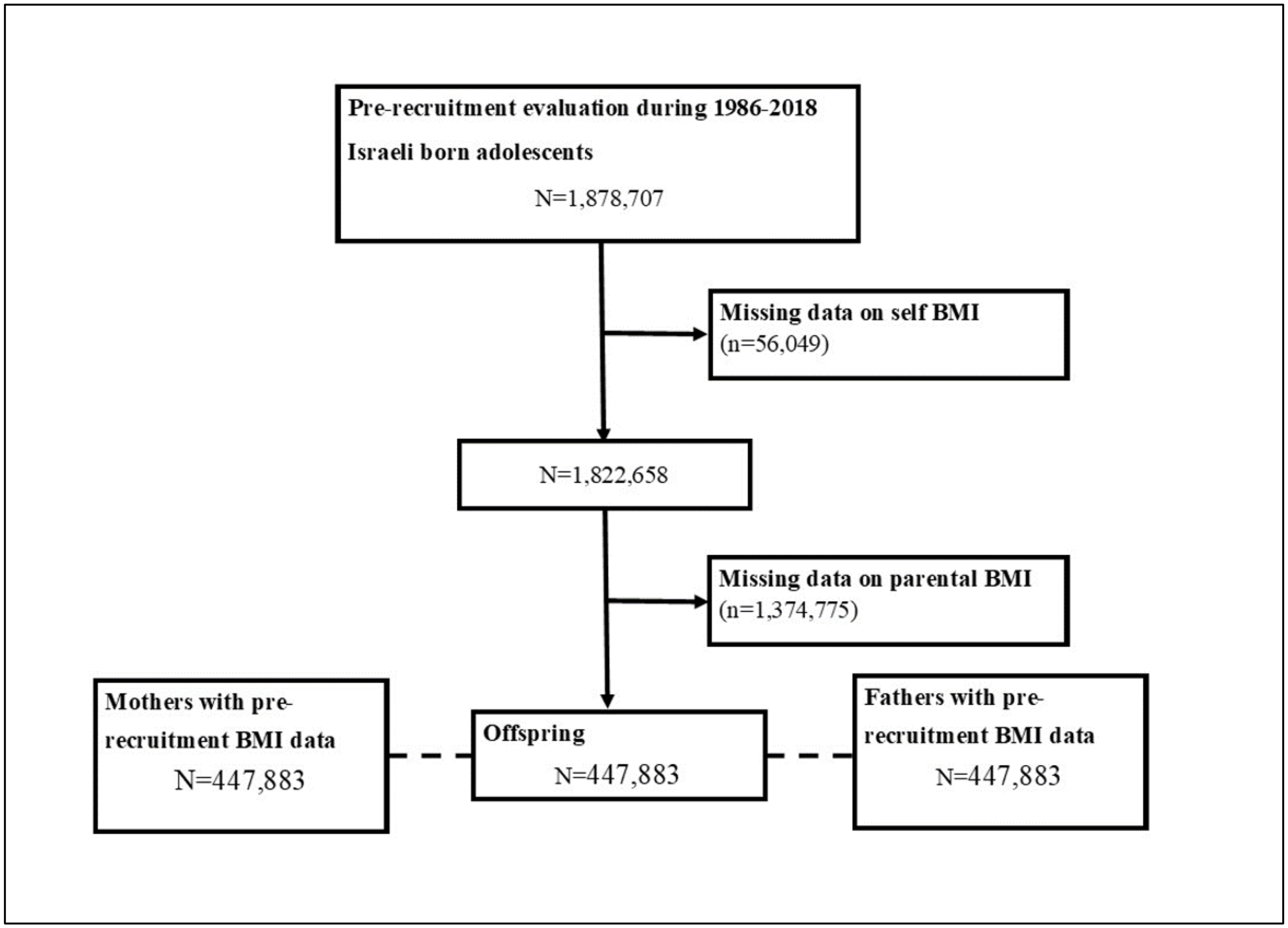

© 2024 Chodick et al. *JAMA Network Open.*

eFigure 2. Odds Ratio for Female Offspring Obesity at Age 17, According to Father's, Mother's, and Mid-Parental BMI Status, Compared to Parent in Normal BMI Range

ORs are adjusted for offspring's age, year of BMI measurement, socioeconomic level, education, cognitive performance, and father's and mother's BMI category.

Category	N (% of total)	OR (95%CI)
**Parent with severe underweight**		
Father	5233 (2.5)	0.38 (0.33-0.45)
Mother	8697 (4.1)	0.294 (0.23-0.38)
Mid parental	6288 (3)	0.16 (0.11-0.22)
**Parent with overweight**		
Father	14813 (7)	2.97 (2.8-3.15)
Mother	13806 (6.5)	3.51 (3.33-3.7)
Mid parental	21266 (10)	3.56 (3.38-3.75)
**Parent with obesity**		
Father	2223 (1)	4.98 (4.6-5.39)
Mother	4576 (2.2)	5.72 (5.15-6.37)
Mid parental	10621 (5)	7.62 (7.22-8.04)

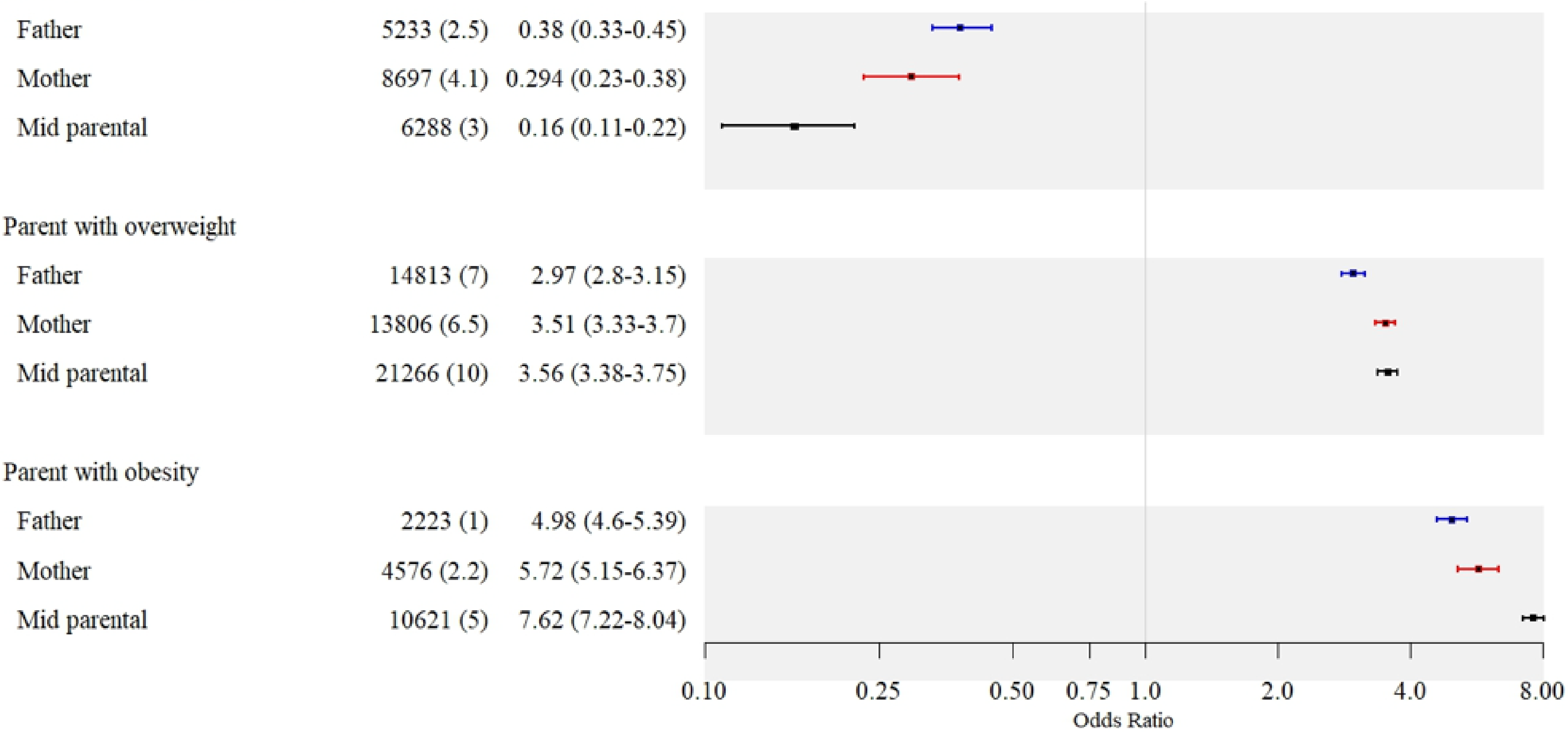

© 2024 Chodick et al. *JAMA Network Open.*

eFigure 3. Odds Ratio for Male Offspring Obesity at Age 17, According to Father's, Mother's, and Mid-Parental BMI Status, Compared to Parent in Normal BMI Range

ORs are adjusted for offspring's age, year of BMI measurement, socioeconomic level, education, cognitive performance, and father's and mother's BMI category.

Category	N (% of total)	OR (95%CI)
Parent with severe underweight		
Father	5913 (2.5)	0.488 (0.439-0.542)
Mother	9988 (4.2)	0.382 (0.327-0.448)
Mid parental	7148 (3)	0.236 (0.196-0.303)
Parent with overweight		
Father	16347 (7)	2.841 (2.712-2.977)
Mother	15197 (6.5)	3.051 (2.95-3.156)
Mid parental	23520 (10)	2.867 (2.752-2.986)
Parent with obesity		
Father	2509 (1.1)	4.148 (3.875-4.441)
Mother	5081 (2.2)	4.487 (4.093-4.919)
Mid parental	11774 (5)	5.759 (5.502-6.028)

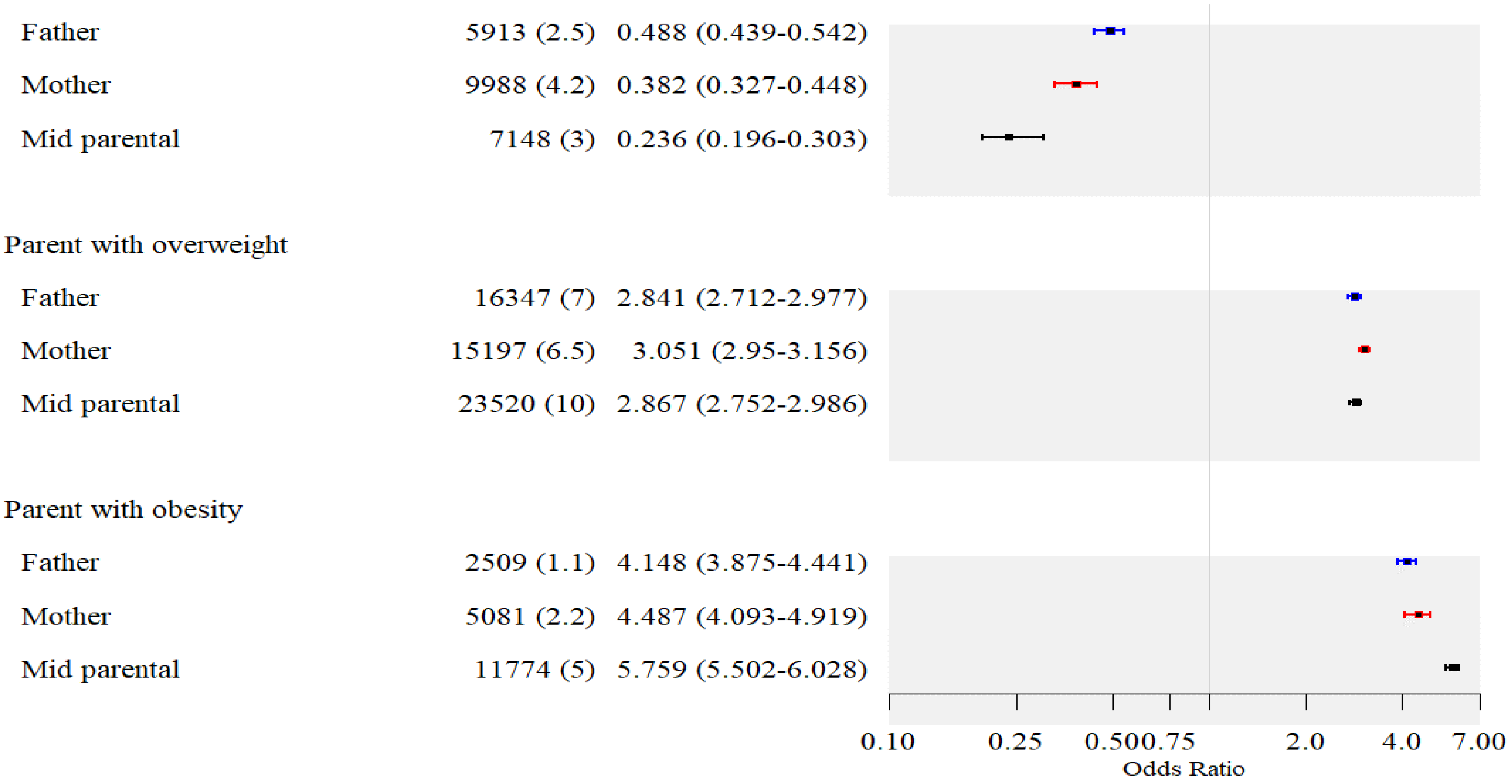

© 2024 Chodick et al. *JAMA Network Open.*

eTable 1. Characteristics of Those Included and Excluded From the Analysis, by Sex and Period

	Men				SMD	Women				SMD
	Excluded		Included			Excluded		Included		
**Examination year**	**(N=298620)**		**(N=27905)**			**(N=222348)**		**(N=25314)**		
**1990-1999**	n	%	n	%		n	%	n	%	
Age, y, M SD	17.3	0.4	17.2	0.3	0.25	17.3	0.4	17.2	0.3	0.26
BMI, m/kg^2, M SD	21.6	3.3	21.6	3.3	0.00	21.6	3.3	21.6	3.3	0.00
Height, cm, M SD	174.3	6.8	175.5	6.7	0.18	162.4	6.1	162.8	6.1	0.07
Weight, kg, M SD	65.6	11.4	66.5	11.4	0.08	57.1	9.8	57.3	9.6	0.02
Unimpaired health	200785	67.2	19426	69.6	0.01	53227	23.9	6313	24.9	0.01
Education, 11+y	271341	91	27124	97.2	0.06	216639	97.8	25099	99.2	0.03
SES										
Low	86890	29.3	3409	12.3	0.41	46854	21.3	2825	11.2	0.27
Medium	147847	49.9	15625	56.3		120199	54.5	14454	57.4	
High	61534	20.8	8714	31.4		53435	24.2	7901	31.4	
Cognitive performance					0.41					0.30
Low	54823	18.4	1899	6.8		28472	12.9	1420	5.6	
Medium	206238	69.4	19699	70.6		173826	78.5	20162	79.6	
High	36317	12.2	6307	22.6		19036	8.6	3732	14.87	
**2000-2009**	**(N=230127)**		**(N=83839)**			**(N=160016)**		**(N=76600)**		
Age, y, M SD	17.3	0.4	17.2	0.3	0.27	17.2	0.4	17.1	0.3	0.27
BMI, m/kg^2, M SD	22.2	3.9	22.1	3.7	0.03	21.9	3.8	21.9	3.7	0.00
Height, cm, M SD	173.8	6.8	174.6	6.7	0.12	161.9	6.2	162.2	6.1	0.05
Weight, kg, M SD	67.1	13.1	67.6	12.6	0.04	57.5	10.9	57.6	10.7	0.01
Unimpaired health	158367	68.8	57075	68.1	0.01	116278	72.7	56789	74.1	0.02
Education, 11+y	215764	93.8	81629	97.4	0.07	156741	98	76014	99.2	0.05
SES										
Low	75694	33	11672	14	0.47	38994	24.4	9677	12.7	0.34
Medium	112271	49	46285	55.3		86692	54.2	42269	55.3	
High	41140	18	25686	30.7		34154	21.4	24497	32	
Cognitive performance										
Low	58327	25.5	10900	13	0.34	33079	20.8	8198	10.7	0.29
Medium	143574	62.7	57144	68.1		113755	71.4	59013	77	
High	27176	11.9	15795	18.8		12429	7.8	9389	12.3	
**2010-2018**	**(N=209187)**		**(N=123192)**			**(N=129450)**		**(N=110735)**		
Age, y, M SD	17.1	0.4	17.1	0.4	0.00	17.1	0.4	17.1	0.3	0.00
BMI, m/kg^2, M SD	22.9	4.3	22.7	4.1	0.05	22.6	4.4	22.4	4	0.05
Height, cm, M SD	173.8	6.8	174.4	6.7	0.09	161.9	6.3	162	6.1	0.02
Weight, kg, M SD	69.1	14.49	69.1	13.7	0.00	59.3	12.5	58.7	11.5	0.05
Unimpaired health	146055	69.8	84889	68.9	0.01	95456	73.7	85292	77	0.04
Education, 11+y	189061	91.1	119516	97.3	0.12	126653	97.8	109562	99.2	0.06
SES										
Low	83633	40.3	19194	15.7	0.58	34780	26.9	15114	13.7	0.34
Medium	93264	44.9	66940	54.6		69103	53.5	60510	54.9	

© 2024 Chodick et al. *JAMA Network Open.*

High	30646	14.8	36363	29.7		25180	19.5	34508	31.3	
Cognitive performance										
Low	54787	26.8	15430	12.5	0.37	26056	20.4	11435	10.3	0.37
Medium	123510	60.3	82385	66.9		90107	70.6	84696	76.5	
High	26338	12.9	25377	20.6		11416	8.9	14604	13.2	

© 2024 Chodick et al. *JAMA Network Open.*

eTable 2. Multivariable Logistic Regression for Obese Offspring According to Parents' BMI

	**Model 1: Age-and-sex adjusted**			**Model 2: Fully adjusted***		
**Father's BMI**	**OR**	**95%CI**		**OR***	**95%CI**	
Severe underweight (n=11146)	0.489	0.448	0.534	0.451	0.413	0.492
Healthy weight (n=400845)	1 (ref)			1 (ref)		
Overweight (n=31160)	2.763	2.665	2.864	2.890	2.786	2.997
Obese (n=4732)	4.410	4.191	4.641	4.482	4.256	4.721
**Mother's BMI**						
Severe underweight (n=18685)	0.364	0.319	0.416	0.352	0.308	0.403
Healthy weight (n=390538)	1 (ref)			1 (ref)		
Overweight (n=29003)	3.247	3.141	3.357	3.079	2.977	3.185
Obese (n=9657)	5.563	5.197	5.956	4.962	4.628	5.319
**Parents' mid-BMI**						
Severe underweight (n=13436)	0.225	0.192	0.265	0.211	0.179	0.248
Healthy weight (n=367266)	1 (ref)			1 (ref)		
Overweight (n=44786)	3.025	2.931	3.121	3.111	3.013	3.212
Obese (n=22395)	6.363	6.149	6.584	6.44	6.22	6.67

* Adjusted for offspring's age, sex, year of BMI measurement, socioeconomic level, education, cognitive performance, and father's and mother's BMI category. **See e-Table 5 for details**

© 2024 Chodick et al. *JAMA Network Open.*

eTable 3. Multivariable Logistic Regression for Offspring With Obesity According to Parents' BMI, Males

	Age adjusted			Fully adjusted*		
**Father's BMI**	**OR**	**95%CI**		**OR***	**95%CI**	
Severe underweight (n=5913)	0.528	0.475	0.586	0.488	0.439	0.542
Normal-range weight (n=210336)	1 (ref)			1 (ref)		
Overweight (n=16347)	2.738	2.615	2.867	2.841	2.712	2.977
Obese (n=2509)	4.101	3.834	4.386	4.148	3.875	4.441
**Mother's BMI**						
Severe underweight (n=9988)	0.393	0.335	0.460	0.382	0.327	0.448
Normal-range weight (n=204839)	1 (ref)			1 (ref)		
Overweight (n=15197)	3.247	3.141	3.357	3.051	2.950	3.156
Obese (n=5081	5.016	4.581	5.491	4.487	4.093	4.919
**Parents' mid-BMI**						
Severe underweight (n=7148)	7148	0.252	0.209	0.303	0.236	0.196
Normal-range weight (n=192663)	1 (ref)			1 (ref)		
Overweight (n=23520)	2.801	2.690	2.916	2.867	2.752	2.986
Obese (n=11774)	5.763	5.484	5.999	5.759	5.502	6.028

* Adjusted for offspring's age, year of BMI measurement, socioeconomic level, education, cognitive performance, and father's and mother's BMI category.

© 2024 Chodick et al. *JAMA Network Open.*

eTable 4. Multivariable Logistic Regression for Offspring With Obesity According to Parents' BMI, Females

	Age adjusted			Fully adjusted*		
**Father's BMI**	**OR**	**95%CI**		**OR***	**95%CI**	
Severe underweight (n=5233)	0.41	0.35	0.48	0.38	0.33	0.45
Normal-range weight (n=190509)	1 (ref)			1 (ref)		
Overweight (n=14813)	2.85	2.69	3.02	2.97	2.80	3.15
Obese (n=2223)	5.00	4.62	5.40	4.98	4.60	5.39
**Mother's BMI**						
Severe underweight (n=8697)	0.30	0.24	0.39	294.00	0.23	0.38
Normal-range weight (n=185699)	1 (ref)			1 (ref)		
Overweight (n=13806)	3.77	3.57	3.97	3.51	3.33	3.70
Obese (n=4576)	6.61	5.95	7.34	5.72	5.15	6.37
**Parents' mid-BMI**						
Severe underweight (n=6288)	0.17	0.12	0.23	0.16	0.11	0.22
Normal-range weight (n=174603)	1 (ref)			1 (ref)		
Overweight (n=21266)	3.48	3.31	3.67	3.56	3.38	3.75
Obese (n=10621)	7.67	7.27	8.09	7.62	7.22	8.04

*Mutually adjusted for offspring's age, year of BMI measurement, socioeconomic level, education, cognitive performance, and father's and mother's BMI category.

© 2024 Chodick et al. *JAMA Network Open.*

eTable 5. Logistic Regression Model: Interaction Terms of Parental BMI Levels

**Unadjusted model**	**OR**	**95% CI**		***P* Value**
Father, normal weight (1)	1(ref)			<0.001*
Father, severe underweight (2)	0.47	0.43	0.52	<0.001
Father, overweight (3)	2.88	2.77	3.00	<0.001
Father, obese (4)	4.61	4.35	4.89	<0.001
Mother, normal weight (5)	1(ref)			
Mother, severe underweight (6)	0.37	0.31	0.42	<0.001
Mother, overweight (7)	3.38	3.26	3.51	<0.001
Mother, obese (8)	5.76	5.32	6.23	<0.001
2 by 6	0.62	0.20	1.94	0.408
2 by 7	1.26	1.01	1.58	0.037
2 by 8	0.87	0.56	1.35	0.523
3 by 6	0.79	0.50	1.25	0.311
3 by 7	0.81	0.73	0.89	<0.001
3 by 8	0.90	0.73	1.10	0.299
4 by 6	1.55	0.97	2.49	0.069
4 by 7	0.82	0.72	0.93	0.002
4 by 8	0.84	0.67	1.05	0.129
**Fully adjusted model****				
Father, normal weight (1)	1(ref)			<0.001*
Father, severe underweight (2)	0.44	0.39	0.48	<0.001
Father, overweight (3)	3.00	2.88	3.12	<0.001
Father, obese (4)	4.63	4.36	4.92	<0.001
Mother, normal weight (5)	1(ref)			
Mother, severe underweight (6)	0.35	0.31	0.41	<0.001
Mother, overweight (7)	3.17	3.05	3.30	<0.001
Mother, obese (8)	5.05	4.67	5.47	<0.001
2 by 6	0.63	0.20	1.99	0.430
2 by 7	1.28	1.02	1.59	0.031
2 by 8	0.88	0.57	1.38	0.586
3 by 6	0.78	0.49	1.25	0.304
3 by 7	0.81	0.74	0.90	0.000
3 by 8	0.89	0.72	1.09	0.257
4 by 6	1.53	0.95	2.47	0.080
4 by 7	0.81	0.71	0.93	0.002
4 by 8	0.87	0.69	1.10	0.249

*P-value for the combined test for interaction between maternal and paternal BMI. ** Adjusted for offspring's age, year of BMI measurement, socioeconomic level, education, cognitive performance, and father's and mother's BMI category.

© 2024 Chodick et al. *JAMA Network Open.*

eTable 6. List of Variables in the Multivariable Logistic Regression Shown in eTable 1

Model 1
**Outcome:**
Offspring with overweight or obesity (BMI ≥85th percentile)
**Exposures:**
Father's BMI category at age 17y
Normal weight, BMI ≥3rd percentile and <85th percentile (ref)
Severe underweight, BMI <3rd percentile
Overweight, BMI ≥85th percentile and <95th percentile
Obese, BMI ≥95th percentile
Mother's BMI category at age 17y
Normal weight, BMI ≥3rd percentile and <85th percentile (ref)
Severe underweight, BMI <3rd percentile
Overweight, BMI ≥85th percentile and <95th percentile
Obese, BMI ≥95th percentile
**Potential confounders:**
Sex (male or female)
Offspring's age at BMI measurement (continuous variable)

Model 2
**Outcome:**
Offspring with overweight or obesity (BMI ≥85th percentile)
**Exposures:**
Father's BMI category at age 17y
Normal weight, BMI ≥3rd percentile and <85th percentile (ref)
Severe underweight, BMI <3rd percentile
Overweight, BMI ≥85th percentile and <95th percentile
Obese, BMI ≥95th percentile
Mother's BMI category at age 17y
Normal weight, BMI ≥3rd percentile and <85th percentile (ref)
Severe underweight, BMI <3rd percentile
Overweight, BMI ≥85th percentile and <95th percentile
Obese, BMI ≥95th percentile
**Potential confounders:**
Sex (male or female)
Offspring's age at BMI measurement (continuous variable)
Calendar year of BMI measurement (continuous variable)
Socioeconomic level
Low, 0-4
Medium, 5-7

© 2024 Chodick et al. *JAMA Network Open.*

High, 8-10 (ref.)
Education
< 11 formal schooling years
11-12 formal schooling years (ref.)
Cognitive performance
<25th percentile
25th-<75th percentile
75th -100th percentile (ref.)

© 2024 Chodick et al. *JAMA Network Open.*
