# Supplementary material for: Heritability of Body Mass Index Among Familial Generations
Source: JAMA Netw Open. 2024 Jun 28;7(6):e2419029. doi: 10.1001/jamanetworkopen.2024.19029 (PMC11214117; doi:10.1001/jamanetworkopen.2024.19029)
Supplement: Supplement 2. — Data Sharing Statement [file jamanetwopen-e2419029-s002.pdf]

Data Sharing Statement

Chodick. Heritability of Body Mass Index Among Multiple Familial Generations. *JAMA Netw Open*. Published June 28, 2024. doi:10.1001/jamanetworkopen.2024.19029

Data
Data available: No

Additional Information
Explanation for why data not available: Restrictions apply to the availability of some or all data generated or analyzed during this study to preserve patient confidentiality or because they were used under license. The corresponding author will on request detail the restrictions and any conditions under which access to some data may be provided.
